# Supplementary material for: How does policy modelling work in practice? A global analysis on the use of epidemiological modelling in health crises
Source: PLOS Glob Public Health. 2025 Jun 6;5(6):e0004675. doi: 10.1371/journal.pgph.0004675 (PMC12143527; doi:10.1371/journal.pgph.0004675)
Supplement: Attachment — Submitted filename: Use of Modelling Article_sb.docx [file pgph.0004675.s003.docx]

**How does policy modelling work in practice? A global analysis
on the use of epidemiological modelling in health crises**

**Authors:** Liza Hadley*^ab^; Caylyn Rich^c^; Alex Tasker^d^; Olivier Restif^a^; Sebastian Funk^b^.
* *Corresponding author (LH667@CAM.AC.UK)*
^a^ *Disease Dynamics Unit, University of Cambridge, Cambridge, UK*
^b^ *Centre for the Mathematical Modelling of Infectious Diseases, London School of Hygiene and Tropical Medicine, London, UK*
^c^ *Independent researcher*
^d^ *University of Bristol, Bristol, UK*

**Abstract:**

This study examines the use of epidemiological modelling by policymakers in response to the COVID-19 outbreak. Prior to COVID-19, there was little readiness for global health systems, and many science-policy networks were assembled as ad-hoc. Moreover, in the field of epidemiological modelling, one with significant sudden influence, there is still no international guidance or standard of practice on how modelled evidence should guide policy during major health crises. Here, we use a multi-country case study on the use of epidemiological modelling in emergency COVID-19 response; to examine the effective organisation of crisis science and policy at different scales. We investigated COVID-19 modelling-policy systems and practices in 13 countries spanning all six UN geographic regions. Data collection took the form of expert interviews with a range of national decision-makers, scientific advisors, and modellers. We examined the use and organisation of modelling in health policy globally , introduced a classification framework for modelling policy systems and recommended its application for future practice. Full analysis and interpretation of the breadth of interview responses were presented, providing evidence for the current and future use of modelling in health crises. We argued that long-standing relationships are fundamental to effective science-to-policy pathways and outlined activities that scientists and policy actors could implement in their countries. This article serves as a first evidence base for the current use of modelling in a recent major health crisis.

**Funding:** Wellcome Trust.
**Key words:** modelling, policy, communication, collaboration, outbreak, decision-making.

**1 Introduction**

This study examined the organisation of epidemiological modelling in COVIDCOVID-19 response in 13 different countries and jurisdictions. Epidemiological modelling was brought to the forefront of public health decision-making during the COVID-19 pandemic; in many countries for the first time. Many countries relied heavily on this type of scientific advice for resource allocation, planning, and forecasting during the pandemic, especially in the face of unprecedented public health interventions and incomplete knowledge[^1–4^](https://www.zotero.org/google-docs/?UPylNd). The uptake of modelling, and more broadly, science advice as a whole, in policy, can be streamlined with improvements to the organisation, translation, and timeliness of scientific evidence. Previous works have identified many open questions[^5,6^](https://www.zotero.org/google-docs/?4VQprO), and it has become clear that best practice is needed for navigating the new prominence of scientific advice in public health decision-making. Our study addressed the *organisation* and *translation* of modelling in policy spheres. We used COVID-19 epidemiology as a case study for health crisis systems and encouraged broader public health researchers to build off this work for navigating best practice discussions with their governments.

As countries have invariably faced differing challenges in COVID-19, a cross-country comparison on the use and organisation of outbreak modelling in policy is vital. It is essential to document these practices such that we can learn from shared experiences. Of most interest are the perceptions of those who were *directly* involved in COVID-19 modelling response - national epidemiological modellers and the decision-makers and scientific advisors with whom they interacted. Particular academic groups have reflected and reported on their own work (for example, Howerton *et al*. in the US, Sherratt *et al*. in the UK[^7,8^](https://www.zotero.org/google-docs/?aqzbN4)), but there is a strong need for a breadth study drawing to gather global practices.

Dedicated research studies on the use and organisation of modelling in outbreak response have previously been sparse. Emergent COVID-19 reflective studies include those run by the interagency COVID-19 Multi-model Comparison Collaboration (CMCC), the KEMRI-Wellcome Trust, and Imperial College London among others[^3,9–12^](https://www.zotero.org/google-docs/?WDRQ2u). In 2020, the CMCC produced early guidance on how models can be used in COVID-19, and set out three distinct classes of policy questions suitable for modelling (projecting epidemiological impact in the absence of intervention, scenario analyses and impact of interventions; modelling-related to costs)[^10^](https://www.zotero.org/google-docs/?PI1c4v). The group also discussed the presentation of results and gave brief examples of the modelling-to-policy infrastructure and collaborations in a few select countries. Further work is now needed to examine modelling-to-policy infrastructures and communication pathways in detail, to provide an evidence base of shared experiences and broader scientific advice in health crises.

Our own study builds on this research gap. Data collection involves in-depth semi-structured interviews with national decision-makers, science advisors, and epidemiological modellers in 13 different countries. Interviewees span all six UN geographic regions and capture the breadth of COVID-19 experience. Our study aims to:

a) Compare national practices on the use and organisation of epidemiological modelling in COVID-19 decision-making.
(b) Present a classification system for the different types of science-policy infrastructure observed.
(c) Propose preliminary recommendations to best practice that are contextual to different country settings.

An adjacent paper considers the topic of communication and visualisation[^13^](https://www.zotero.org/google-docs/?7mMSw3).

Below, we reported a qualitative analysis of our findings, using thematic analysis and ideal-type analysis[^14,15^](https://www.zotero.org/google-docs/?SN9Uxk).

**2 Methods**

**2.1 Country and Participant Selection**

This study defines epidemiological modelling activity in a broad sense, including statistical modelling and analytics too. In total, 12 countries and 1 jurisdiction (Hong Kong, China) participated in the study. For brevity, we use the term ‘country' to mean ‘country or jurisdiction' throughout this article, with the knowledge that Hong Kong, China, is a jurisdiction or ‘special administrative region’ and not itself an independent country. Stratified random sampling by UN geographic region was used to select the first country in each region; the remaining countries were chosen to represent the breadth of COVID-19 experience, capturing LMICs and HICs, low- and high- COVID-19 prevalence, and a mix of prior knowledge of modelling for national decision-making (or none), formal modelling structures or consortiums (including those with no formal capacity), pre-COVID-19 pandemic preparedness ratings, and population size. Researchers then used purposeful sampling to contact key modelling-to-policy actors in each country for interview[^16^](https://www.zotero.org/google-docs/?RmrWdV). If no relevant participants could be sought, a second country was chosen. Countries with a population of less than 5 million were excluded. In total, 27 participants from 13 countries took part. Recruitment ran from 21^st^ April 2023 to 10^th^ May 2024.

Participant names are kept anonymous, but participants reported the following as their countries (or jurisdictions) of practice: Australia, Canada, Colombia, France, Japan, Hong Kong Special Administrative Region of the People’s Republic of China (Hong Kong), Kenya, New Zealand, Peru, Republic of Korea (South Korea), South Africa, Uganda, and the United Kingdom. At least two respondents were recruited from each of the above countries. Table M1 describes the countries in our study and Table M2 describes the make-up of participants. Of the 27 interviewees, 12 self-identified as scientific advisors or policy/ decision makers (3, 9 respectively), and 15 as modellers. The group of scientific advisors and decision-makers included national Government Chief Scientific Advisors and heads of the relevant national Health department or agency, as well as intermediaries or convenors assigned specifically to coordinate or relay modelling activities.

| **Country of practice** | **Country details** | | | |
| --- | --- | --- | --- | --- |
|  | **UN geographic region^a^** | **Income status^b^** | **GHS pandemic preparedness rating (2019)^c^** | **Population size (millions)^d^** |
| Australia | Oceania | HIC | 4th | 25.8 |
| Canada | Northern America | HIC | 5th | 38.0 |
| Colombia | LAC | UMIC | 65th | 51.2 |
| France | Europe | HIC | 11th | 64.5 |
| Hong Kong, China | Asia | HIC | 51st* | 7.5 |
| Japan | Asia | HIC | 21st | 124.9 |
| Kenya | Africa | LMIC | 55th | 52.5 |
| New Zealand | Oceania | HIC | 35th | 5.1 |
| Peru | LAC | UMIC | 49th | 33.5 |
| South Korea | Asia | HIC | 9th | 51.8 |
| South Africa | Africa | UMIC | 34th | 59.1 |
| Uganda | Africa | LIC | 63rd | 45.1 |
| United Kingdom | Europe | HIC | 2nd | 67.2 |

*Table M1: The 13 countries/ jurisdictions involved in our study and relevant demographic statistics. Each interviewee reported their country(ies) of practice as one of the above. LAC = Latin America and the Caribbean; LIC = Low-income country; LMIC = Lower-middle income country; MIC = Upper-middle income country; HIC = High-income country. Country data is taken from the following sources: (a) & (b) UN geographic region, country income, and population (as of 1^st^ January 2021) is as described in the United Nations World Population Prospects 2022*^17,^[^22^](https://www.zotero.org/google-docs/?QupDHj); *(c) Rankings taken from the 2019 Global Health Security Index, indicating expected overall preparedness for an infectious disease outbreak^18^. Note that Hong Kong’s ranking of 51st represents the overall ranking for China - as noted in the main text, Hong Kong is a Special Administrative Region of China.*

| **Affiliation** | **Role** | **No. of interviewees** |
| --- | --- | --- |
|  |  |  |
| Academic | Modeller | 15 |
|  | Independent scientist and advisor | 3 |
| Government | Policymaker | 5 |
|  | Senior government (For example head of a Ministry of Health or Public Health Agency, senior Chief Scientific Advisors, senior members of Cabinet, and related roles) | 4 |

*Table M2: Make-up of study participants.*

**2.2 Interview format and location**

Interviews were in-depth and semi-structured, lasting approximately 1 hour each. The format was a structured discussion on four topics in turn, facilitated by the interviewer. Additionally, the first interviewee in each country was invited to co-create a ‘sketch diagram’ with the interviewer, mapping out the key actors in modelling and policy in their country from their perspective. This rough diagram served as a reference point for discussion, and the main pathways were verbally verified through discussion with subsequent interviewees. The remainder of the interview followed the standard semi-structured format.

The interview schedule was heavily informed by previous work on ‘Challenges for Future Pandemics’ in 2020/2021. Hadley *et al.*, 2021 introduce a succinct framework that defines the areas for progress on the interface of outbreak modelling and policy (reproduced here as Figure M1)[^6^](https://www.zotero.org/google-docs/?QmWfgy). This idea-generating piece directly informed our study’s core themes and specific interview questions. Hadley *et al.*, 2021 identified seven broad challenges of pandemic modelling for policy; we narrowed this down to focus on four related interviews ‘themes’, namely ‘Structures and pathways to policy’ (relating to challenges 1 & 3 in Figure M1), ‘Collaboration and knowledge transfer’ (2&4), ‘Communication and visualisation’ (5), and ‘Evaluation and reflection’ (7). Our study did not discuss technical model challenges, as this has been frequently researched and reported on in the broader literature (as one example, see Sherratt *et al*. discussing European nations[^19^](https://www.zotero.org/google-docs/?0lTVqs)). The full interview schedule was included in the Supplementary Material. Results on visual preferences were presented in our adjacent study[^13^](https://www.zotero.org/google-docs/?C6M5g0). Four interviews were conducted in person; the remaining 23 were conducted online via video-conferencing software. Majority of interviews were conducted in English, with one using an independent translator to aid communication.

*Figure M1: Infographic depicting seven broad challenges of pandemic modelling for policy around the central equation of epidemic spread. Reproduced from Hadley et al., 2021*[*^6^*](https://www.zotero.org/google-docs/?nsXips)*.*

**2.3 Data collection and analysis**

All interviews were audio recorded, transcribed, independently ‘coded’, and then reviewed for accuracy by the study researchers. Data analysis followed a two-pronged approach: using thematic analysis for descriptive findings and ideal-type analysis for a more in-depth understanding of the data, enabling contextual recommendations to be made on the future of modelling[^14,15^](https://www.zotero.org/google-docs/?GSgWdQ). A draft version of this article was also circulated to all interviewees to enable verification and opportunity for revisions of the key findings.

Results sections 3.1 and 3.2 present key data from each country and introduce a classification framework for modelling policy systems in health crises. Section 3.3 examines any collaboration of modelling with other scientific disciplines, section 3.4 explores translators and knowledge brokers, and section 3.5 discusses mistakes. Finally, section 3.6 draws on the complete study analysis, supplementary results, and pre-existing literature to introduce guidelines for future practice on using modelling in health crises.

**2.4 Ethics statement**

The University of Cambridge Psychology Research Ethics Committee (application number PRE.2023.034) has reviewed this research. All participants provided informed written consent.

**3 Results**

Results on the current use of modelling (3.1-3.5) and recommendations for future practice (3.6) were presented here. Additional findings on communication and visualisation were presented in the adjacent paper by Hadley *et al*.[^13^](https://www.zotero.org/google-docs/?cg1ASz).

**3.1 Organisation of modelling infrastructure**

Our analysis identified two key parameters concerning the modelling pathway to national decision-making within each country: size/type of modelling infrastructure, and level of government interaction with modelling. We observed four distinct types of national **modelling** **infrastructure**, presented in Figure R1. These are one modelling team, multiple small teams functioning as one, a consortium consisting of multiple teams and multiple models led by a modelling committee, and finally, teams working in isolation, feeding independently into government. Types 2 and 3 were separated to distinguish a single-model infrastructure that would likely have no capacity to work on the same questions from a multi-model infrastructure that does have the capacity for self-verification, reproduction, or consensus (in any form). Type 4 considers settings where modelling activity is disjoint; modellers likely do not confer with each other, and results are instead ‘combined’ in-house by a government non-modelling committee. Examples were given in Figure R1. Our classification aims to capture the size of each country’s primary modelling capacity, ability or inability to reproduce model findings, and whether modelling outputs are *synthesised and interpreted by modellers* (1-3) or *non-modellers* (4) such as other infectious diseases or policy experts.

*Figure R1: The four types of national modelling infrastructure observed in our study. One modelling team (1); multiple small teams functioning as one (no capacity to reproduce) (2); multiple teams and multiple models (capacity to reproduce) led by a committee of modellers (3); modellers and/or modelling teams do not collaborate and feed results directly into government to be combined by a committee of non-modellers (4).* Pictured: Individuals with hats represent modelling principal investigators/ senior modellers. Computers represent models. Boxes represent a modelling committee. For example, in South Africa (type 2), there was insufficient capacity to examine the same modelling questions with multiple models, so teams divided the research questions among themselves. In South Korea (type 4), the modelling effort was disjointed - modelling teams did not collaborate or discuss findings and instead sent reports directly to the relevant policy actor.

Additionally, one can classify each country by the **level of government interaction in modelling**. This is shown in Table R1 (rightmost column), along with summary findings on each country’s structures and pathways to policy. The leftmost columns concern *modelling* infrastructure, while the rightmost columns consider the policy infrastructure for modelling and provide a relative ranking for the level of government interaction and independence in each setting, based on in-depth discussions with interviewees. One sees, for example, that the level of interaction in modelling from government bodies varied greatly (Medium: 3 countries; High: 5; Very high: 2; Embedded: 2). Illustrative definitions of these rankings are provided in the Supplementary Material. Countries where modelling had little to no use by the national government in COVID-19 were excluded from our study.

| **Country** | **Type of modelling infrastructure (type #)** | **Name of MC or team if given** | **Main affiliation of modellers** | **Main government linkage point(s)** | **Level of (structural) government interaction** |
| --- | --- | --- | --- | --- | --- |
| ***Africa*** | | | | | |
| Kenya | Multiple teams, multiple models (3) | Led by Centre for Epidemiological Modelling and Analysis (CEMA), University of Nairobi | Universities or research institutes | Health, also Education, Faith  *Multiple linkages reported: MoH mainly, also Ministry of Education, National Council of Churches and religious groups, National Taskforce, and Cabinet at times* | High. Kenyan COVID-19 modelling review team sits within the National COVID-19 Task Force. |
| South Africa | Multiple teams, one model (2) | South African COVID-19 Modelling Consortium (SACMC) | Universities or research institutes | Health, Economics  *Ministry of Health advisory groups, MoH direct at times, Treasury* | High. SACMC convened by the National Institute of Communicable Diseases at the request of MoH. |
| Uganda | Teams working independently, feeding into government (4) | NA | Universities or research institutes | Health  *Epidemiological Advisory Group, PRESIDE and National Planning Authority* | Medium. Two modellers on relevant SACs. |
| ***Asia*** | | | | | |
| Hong Kong, China | One team (1) | Led by LKS Faculty of Medicine, University of Hong Kong researchers | Universities or research institutes | Health  *COVID-19 Expert Advisory Panel, and Cabinet at times* | High. Highly-integrated key individuals as brokers. |
| Japan | One team (1) | Led by Kyoto University researchers | Universities or research institutes | Health  *Ministry of Health* | Very high. Modellers in-housed to MoH during first wave and modeller on relevant SAC. |
| South Korea | Teams working independently, feeding into government (4) | NA | Universities or research institutes | Health  *Korean Disease Control and Prevention Agency (CDC)* | Medium. Brokerage carried out by influential ID specialist due to limited modelling expertise. |
| ***Europe*** | | | | | |
| France | One team initially (1); then multiple teams, multiple models (3) | Led by Institut Pasteur researchers; later the Coordinated Action of Modelling for Disease (ACM) | Universities or research institutes | Health  *Conseil Scientifique* | Medium. One modeller on national SAC throughout. |
| United Kingdom | Multiple teams, multiple models (3) | Scientific Pandemic Influenza Group on Modelling, Operational (SPI-M-O) | Universities or research institutes primarily, with government contributors | Health primarily  *SAGE (Government Office for Science); Cabinet Office at times* | High. Several modellers on national SAC throughout, secondments to Cabinet Office, and regular attendees from Cabinet Office to MC meetings. |
| ***Latin America and the Caribbean*** | | | | | |
| Colombia | Multiple teams, multiple models (3) | Led by National Institute of Health (INS) | Government, with university/ research institute teams contributing on occasion | Health, Education  *Ministry of Health, Ministry of Education* | Embedded (primary modelling team). |
| Peru | One team (1) | CDC-Peru modelling unit | Government | Health  *Ministry of Health* | Embedded. |
| ***Northern America*** | | | | | |
| Canada | Multiple teams, multiple models (3) | Led by the Public Health Agency of Canada (PHAC) | Government, with university/ research institute teams contributing regularly | Health  *Minister of Health, Cabinet* | Embedded (primary modelling team). |
| ***Oceania*** | | | | | |
| Australia | One team (scenarios) (1); multiple teams, multiple models (forecasting) (3) | Doherty Modelling consortium and National COVID-19 Situational Assessment Consortium | Universities or research institutes primarily | Health, Economics  *Multiple linkages including Australian Health Protection Principal Committee (AHPPC), Treasury* | High. Long-term pre-existing relationship and modellers on multiple SACs. |
| New Zealand | Multiple teams, multiple models (3) | COVID-19 Modelling Aotearoa (CMA) | Universities or research institutes | Health  *Ministry of Health, Department of Prime Minister and Cabinet* | Very high. Extremely close science-policy system but still with modellers’ independence. |

***Table R1: Modelling infrastructure and details on the structures and pathways to policy in each country in COVID-19, as described by interviewees. MC = Modelling Committee. SAC = A multidisciplinary Scientific Advisory Committee or expert panel. MoH = Ministry of Health / Department of Health. ID = Infectious Diseases.***

**3.2 Types of modelling-to-policy systems**

It is important to identify countries with similar experience in relation to the use of modelling to guide future recommendations. As noted in Section 3.1, the size/type of modelling capacity and level of government interaction with modelling were identified as key contexts to consider when grouping country settings. In Figure R2, we present a classification of countries with similar beliefs and experiences in outbreak modelling and policy. This is called a ‘typology’[^15^](https://www.zotero.org/google-docs/?2BCF3S) in qualitative research (see Supplementary Material for further details and a derivation). Five categories of modelling-policy systems were identified: (A) Countries with small modelling capacity and likely high government linkage; (B) Countries with large modelling capacity and high or very-high government linkage; (C) Countries where modelling teams worked independently, combined by non-modellers in government; (D) Countries with a large modelling capacity with a primary government modelling team; (E) Countries with one government modelling team.

*Figure R2: Categories of modelling-policy systems. Size of modelling capacity and level of government interaction with modelling were identified as key contexts to consider when grouping country settings. The five categories of modelling-policy systems identified are: (A) Countries with small modelling capacity and likely high government linkage, such as Hong Kong; (B) Countries with large modelling capacity and high or very high government linkage, such as Kenya; (C) Countries where modelling teams worked independently, combined by non-modellers in government, such as South Korea; (D) Countries with a large modelling capacity with a primary government modelling team, such as Canada; (E) Countries with one government modelling team, such as Peru. Countries were placed in the same category if interviewees demonstrated similar beliefs and experiences on the use of modelling in policy. Note: For other pathogens and other health crises, each category may contain different countries.*

Producing a robust categorisation of current modelling-policy systems as shown in Figure R2 facilitates context-specific recommendations for future practices. To this end, these categories present subsequent results where it is meaningful to do so.

**3.3 Collaboration with other disciplines**

In most cases, modelling activity was focussed on primary health outcomes (i.e. COVID-19 transmission and disease), but there were a few interdisciplinary success stories where modellers collaborated with other disciplines. Costing, or the idea of evaluating trade-offs (either health or economic-based), was reported by modellers in New Zealand, Australia, Canada, and Peru but was explicitly indicated as not existent in the UK, Colombia, and France. In New Zealand, while economic modelling remained the responsibility of the Treasury, the country’s modelling consortium (COVID-19 Modelling Aotearoa, CMA) collaborated to provide analyses of isolation policy trade-offs and linked informal health modelling within the CMA to economic modelling within the Treasury.

On the behavioural sciences front, in Australia, modellers previously collaborated with social scientists on the use of citizen juries to inform ethical vaccine allocation, but during the COVID-19 response, such networks suffered a lack of behavioural social sciences inclusion. A respondent from South Korea also emphasised the importance of an interdisciplinary approach to modelling: “This is a policy where extensive educational, social, and economic losses are expected, so it is necessary to listen to the opinions of experts in each field.” Different forms of science can of course be combined ‘higher up’ in senior government, but there was a shared sentiment of desire for more cross-pollination in countries that lacked it. For example, post pandemic, modellers from Kenya reported that they are now actively working with behavioural scientists in a local School of Anthropology on the current Rift Valley Fever outbreak.

Note: Limitations to the scope of this analysis resulted in a lack of evaluation for the *quality* of collaboration across disciplines, but rather sought to determine whether collaboration existed.

**3.4 Translators and knowledge brokers**

We define ‘translators’ as those individuals who took on the role of packaging information, converting complex epidemiological modelling findings into meaningful language for policy. In countries where modellers collaborated and had a level of independence from government (categories A and B in Figure R2), this ‘translator’ role was often taken on by the most senior modeller(s) (Australia, France, Kenya, South Africa) or epidemiologist (Hong Kong). Exceptions include New Zealand’s small close-knit science-policy ecosystem where modellers interacted directly with senior government and first translation was performed largely by the relevant Chief Scientific Advisor, and the UK whose modelling committee (SPI-M-O) was jointly convened by a policy co-chair and academic co-chair. In the latter, the policy co-chair and SPI-M-O secretariat were all civil servants. Uniquely these UK individuals had significant past modelling expertise and could effectively synthesise key modelling findings that the policy co-chair, crucially *inside* government, could take into the parts of government that the academics reportedly had limited access to. Secretariat members ensured that an accurate interpretation of the modelling was relayed to ministers by meticulously agreeing appropriate slide headings with Cabinet Office officials to ensure the knowledge was conveyed appropriately; the benchmark became whether policy makers would be able to understand the implications of the content if they only read the headings.

Countries with more government embeddedness (categories D and E in Figure R2) saw a mix of translators: the Chief Public Health Officer in Canada, the lead academic modeller in Japan, and the relevant Peru CDC department director in Peru. Finally, in South Korea (category C - disjointed modelling) where there were reported to be no dedicated infectious disease modelling teams at the beginning of the COVID-19 pandemic (primarily mathematics or public health research groups with only some modelling expertise), translation was taken on by an experienced and informed non-modelling infectious diseases principal investigator. The origin of each of the above interactions varied; as one interviewee commented “it is hard to create strict rules around who should communicate: it ends up being the persons involved where their strengths lie and what structures they have around them”.

**3.5 Mistakes**

Many respondents were asked what they perceived to be the ‘biggest mistake’ when using modelling in the response. Responses are shown in Table R2 for interest. These comments were shared with the interviewer in the spirit of identifying shared challenges and should be interpreted not as shortcomings of a particular individual but as areas where we could collectively improve.

**What was your biggest mistake (in relation to the use of modelling)?**

Not being transparent enough with lack of knowledge; allowing policy makers to espouse more certainty in the models than existed

Inability to address vaccine hesitancy - lacked incorporating behavioural or economic scientists into modelling which contributed to elevated vaccine hesitancy

Underestimating the number of deaths

Relying on a single model/ not managing to develop a multiple model consortium

Lack of understanding of types of modelling and modelling’s limitations (not investing in the pedagogy of modelling)

Not taking an interdisciplinary approach, only collaborating with the Ministry of Health rather than other ministries

Utilising worst-case scenarios to justify actions, especially when presented in a public forum

Erring too far on the pessimistic side of modelling/ the negative outcome or worst-case scenario

Accidentally getting too involved with politically motivated media figures

Lack of resources (financial) to pool expertise

Risk in using models as the primary reason for introducing a lockdown

Not believing vaccines would be available so quickly

Not grasping the impact of interventions

Lack of resourcing creating biases in datasets - marginalised communities couldn't enjoy same level of for example surveillance consequently forcing ‘national’ rather than ‘local’ models onto indigenous communities

Some modellers/ modelling groups struggled to recognize the time and place appropriate for complex vs simple models

Narrow scope of metrics, no modelling of socioeconomic impacts

Downplaying models’ predictions

“I don’t think we made a mistake but with hindsight we’ve always thought, thank God, we kind of did it right and we didn’t get it wrong”

Not being prepared capacity-wise

Poorly communicating the risks

***Table R2: Responses to the question “What was your biggest mistake (in relation to the use of modelling)?”. All responses have been paraphrased to maintain anonymity.***

**3.6 Recommendations for future practice**

Towards the end of each interview, interviewees discussed their key takeaways and recommendations for the future of outbreak modelling and policy. From the results of our study analysis, recommendations from interviewees (presented in full in Supplementary Material S5), and additional literature in this area, we introduce the following guidelines for future practice on using modelling in health crises:

| **Guidelines for future practice on using modelling in health crises** | | |
| --- | --- | --- |
|  |  |  |
| **1** | **Identify your system** | There is no optimal modelling-policy system. Moving between categories of modelling-policy systems may require significant political and cultural change. Scientists should instead identify the system in which they operate (Figure R2) and utilise the context-specific case studies and guidance presented in this article. |
| **2** | **Relationships** | Facilitate long-standing relationships. Consider developing relationships with government groups rather than individuals, to preserve future linkages under government turnover. For systems where modelling sits primarily ‘outside’ government, it is critical to have an interpreter/translator or advocate (who may or not be a modeller) within policymaking spheres to provide a space for modelling. |
|  |  |  |
|  |  |  |
| **3** | **Standing capacity for modelling** | More standing capacity for modelling is needed in some countries. For systems with limited modelling capacity, provide secure jobs for modellers. Disjointed modelling effort is encouraged to unify informally. Where there is a lack of capacity to train people in-country, an international modelling network or society could aid development. Large modelling capacity and multi-model consortiums will likely need to be maintained with dedicated funding; where that exists we encourage active sharing of ideas to countries with fewer experts. |
| **4** | **Data availability** | Strong data access and availability is essential for modelling. This is a universal recommendation for all settings. |
| **5** | **Legitimacy** | For settings where modelling sits ‘outside’ of government, establish relationships and agreements/ memorandums of understanding ahead of time. Unifying modelling effort through a modelling committee can aid identifiability in policy spheres. Retrospective verification of modelling results is also important for developing trust and legitimacy. |
| **6** | **Communicating modelling** | Caution against overbelieved or misinterpreted modelling. For detailed guidance on communicating and interpreting modelling in policymaking, see adjacent study[^13^](https://www.zotero.org/google-docs/?Up1NC1). |
| **7** | **Interdisciplinarity** | Science advice in health crises should be holistic, looking beyond direct health outcomes. However, research on how modelling can best interact with economics and behavioural sciences is limited. We offer examples (Section 3.3) but further research is needed before a formal guideline can be made here. |

***Table R3: Preliminary guidelines for future practice on using modelling in health crises. From the results of our study analysis, recommendations from interviewees, and additional literature in this area, we suggest the following guidelines for future practice on using modelling in health crises.***

**4 Discussion**

At the time of shock, when the pandemic first hit, nations organised scientific and modelling capacity, and crucially this self-organisation happened in *different* ways, resulting in *different successes and challenges* as outlined in detail above. More universally, there was a desire for modelling to have an eye to wider societal impacts, and in many settings, work formally with other experts. We highlight two success stories of modelling successfully working with economics and social science either in the COVID-19 pandemic or as an active post-COVID-19 advancement.

On the communication front, we introduced the idea of *re-translation*, acknowledging the need to ensure that not only is modelling translated and packaged effectively to policymakers, but that in subsequent iterations ‘up the chain’ the message is maintained. There is a need now for further emphasis in Science Communication work on enabling *subsequent* translation of modelling, at least in policy settings.

Finally, we acknowledged overarching themes of data, communication, systems, and relationships as primary enablers to successful evidence-based practice. We synthesised specific and contextual recommendations for future practice on the use of modelling in health crises. These stem from crucial suggestions from global key actors, many with extensive experience in multiple health events and from different perspectives. This work provides a first evidence base to begin best practice agreements in the newly high-profile field of public health modelling.

Looking at related literature in this area, Owek *et al*.^3^identified pre-existing relationships between researchers and decision-makers as an enabling factor for successful knowledge translation, and co-creation of advice and embeddedness[^3^](https://www.zotero.org/google-docs/?ORZG2t). Our findings echo this, with ‘Systems’ and ‘Relationships’ both being pivotal for future outbreak modelling and response. Our findings on systems also reflect that of formal inquiries taken on by several countries - for example, in the UK’s COVID-19 Inquiry, modellers (as well as all other scientists, policymakers, decision-makers, and associated key actors) provided witness statements detailing their involvement in the country’s pandemic response from which it is clear that the systems and relationships built before and during COVID-19 were fundamental to modelling’s utility in the pandemic response[^20^](https://www.zotero.org/google-docs/?1TDwnb).

Additionally, there was an acknowledgement of the role of translators by the CMCC discussed earlier in this article, noting that where needed, “decision-makers may be supported by advisors or knowledge brokers with a high literacy to interpret models”. In contrast, Owek *et al*.^3^ found that face-to-face debriefings between scientists and policymakers were preferred in Low- and Middle-Income Countries over the use of a knowledge broker or intermediary. We observed more of a mix of translators in our study - of the five countries classified as LMICs in Table M1 (Colombia, Kenya, Peru, Uganda, and South Africa), translation was taken on by the most senior modeller in three settings but by an intermediary in the other two. It is worth acknowledging whether academics should be the ones responsible for making strong visuals or if this activity could be more effectively in-house. There is merit for the latter in the countries we have investigated.

Regarding limitations, we have aimed to capture a range of experiences in our study. However, there was a limited number of participants (27) and in most cases, only two participants per country. Consequently, we did not attribute recommendations to specific countries (with the exception of giving examples) and instead presented the major analyses by typology (e.g., suggestions for countries with small modelling capacity, suggestions for countries where modelling sits ‘outside’ of government, etc.). Note also that interview data represents the *perceptions* of interviewees. Hence, this study only evaluates the perceived use of modelling, albeit from highly informed individuals. Nonetheless, this may not reflect official policy or how individuals would behave in practice. We also use the term ‘government’ loosely throughout and acknowledge that this relates to different individuals and committees in each country due to the multifaceted political structures.

We also acknowledge that state-level and regional (multi-country) modelling were outside the scope of this study, as were case study countries where modelling *did not* contribute to national COVID-19 response. State-level modelling played a role in some of the larger countries of our study, such as Colombia, Canada, and Australia, but these activities are not reported here.

With the above limitations in mind, our study provides an evidence base for the use of modelling in different countries during a major recent health crisis (COVID-19), and analysis has enabled initial recommendations from which further research can build. This study will directly contribute evidence to formal activities to develop best practices, such as the Lancet Commission on ‘Strengthening the Use of Epidemiological Modelling of Emerging and Pandemic Infectious Diseases’[^21^](https://www.zotero.org/google-docs/?TmnTY7).

**Author contributions**

LH proposed the study. LH, OR, AT, and SF were involved in conception and design. LH carried out the data collection. LH, CR, and AT carried out analysis and interpretation of results. LH wrote the first draft of the manuscript. All authors provided feedback on the manuscript and approved the final version for publication.

**Acknowledgements**

LH acknowledges support from the Wellcome Trust (block grant no. RG92770). SF also acknowledges support from the Wellcome Trust (grant no. 210758/Z/18/Z). The authors also thank Sungmok Jung, Mircea Sofonea, and all other interviewees for dedicating their time and experience to the study.

The authors declare no conflicts of interest.

**Data availability**

The available anonymised data has been presented in this manuscript and any associated manuscripts where possible.

**References**

[1.	Aguas R, White L, Hupert N, Shretta R, Pan-Ngum W, Celhay O, et al. Modelling the COVID-19 pandemic in context: an international participatory approach. BMJ Glob Health. 2020 Dec 23;5(12):e003126.](https://www.zotero.org/google-docs/?Td9zY9)

[2.	Jit M, Ainslie K, Althaus C, Caetano C, Colizza V, Paolotti D, et al. Reflections On Epidemiological Modeling To Inform Policy During The COVID-19 Pandemic In Western Europe, 2020–23. Health Aff (Millwood). 2023 Dec;42(12):1630–6.](https://www.zotero.org/google-docs/?Td9zY9)

[3.	Owek CJ, Guleid F, Maluni JK, Jepkosgei J, Were V, Sim SY, et al. LESSONS LEARNED FROM COVID-19 MODELLING EFFORTS FOR POLICY DECISION-MAKING IN LOWER- AND MIDDLE-INCOME COUNTRIES [Internet]. 2024 [cited 2024 Mar 13]. Available from: http://medrxiv.org/lookup/doi/10.1101/2024.01.30.24301978](https://www.zotero.org/google-docs/?Td9zY9)

[4.	Xiang Y, Jia Y, Chen L, Guo L, Shu B, Long E. COVID-19 epidemic prediction and the impact of public health interventions: A review of COVID-19 epidemic models. Infect Dis Model. 2021 Jan 1;6:324–42.](https://www.zotero.org/google-docs/?Td9zY9)

[5.	Mollison D, Isham V, Dangerfield C, Hollingsworth D. Preface: Challenges for future pandemics. Epidemics. 2022 Sep 1;40:100621.](https://www.zotero.org/google-docs/?Td9zY9)

[6.	Hadley L, Challenor P, Dent C, Isham V, Mollison D, Robertson DA, et al. Challenges on the interaction of models and policy for pandemic control. Epidemics. 2021 Dec 1;37:100499.](https://www.zotero.org/google-docs/?Td9zY9)

[7.	Howerton E, Contamin L, Mullany LC, Qin M, Reich NG, Bents S, et al. Evaluation of the US COVID-19 Scenario Modeling Hub for informing pandemic response under uncertainty. Nat Commun. 2023 Nov 20;14(1):7260.](https://www.zotero.org/google-docs/?Td9zY9)

[8.	Sherratt K, Carnegie AC, Kucharski A, Cori A, Pearson CAB, Jarvis CI, et al. Improving modelling for epidemic responses: reflections from members of the UK infectious disease modelling community on their experiences during the COVID-19 pandemic. Wellcome Open Res. 2024 Jan 8;9:12.](https://www.zotero.org/google-docs/?Td9zY9)

[9.	CMCC Technical Group. Technical Group Report: Model Fitness-for-purpose Assessment Report [Internet]. 2020 Aug. Available from: https://decidehealth.world/sites/default/files/2020-09/Technical_Report_Sept1.pdf](https://www.zotero.org/google-docs/?Td9zY9)

[10.	CMCC Policy Group. Guidance on Use of Modelling for Policy Responses to COVID-19 [Internet]. 2020 Aug. Available from: https://decidehealth.world/sites/default/files/2020-09/Policy_Report_Sept.1.pdf](https://www.zotero.org/google-docs/?Td9zY9)

[11.	Oliwa JN, Guleid F, Owek CJ, Maluni J, Jepkosgei J, Sim SY, et al. A FRAMEWORK TO GUIDE THE USE OF MATHEMATICAL MODELLING IN EVIDENCE-BASED POLICY DECISION-MAKING. 2024.](https://www.zotero.org/google-docs/?Td9zY9)

[12.	Christen P, Van Elsland S, Saulo D, Cori A, Fitzner J. Workshop Report: Advanced analytics to inform decision making during public health emergencies [Internet]. WHO Hub for Pandemic and Epidemic Intelligence, Berlin, Germany: [object Object]; 2023 May [cited 2024 Apr 15]. Available from: http://spiral.imperial.ac.uk/handle/10044/1/108600](https://www.zotero.org/google-docs/?Td9zY9)

[13.	Hadley L, Rich C, Tasker A, Restif O, Funk S. Visual preferences for communicating in crises: evidence from public health decision makers [Internet]. medRxiv; 2024 [cited 2024 Dec 16]. p. 2024.11.05.24316774. Available from: https://www.medrxiv.org/content/10.1101/2024.11.05.24316774v2](https://www.zotero.org/google-docs/?Td9zY9)

[14.	Braun V, Clarke V. Using thematic analysis in psychology. Qual Res Psychol. 2006 Jan 1;3(2):77–101.](https://www.zotero.org/google-docs/?Td9zY9)

[15.	Stapley E, O’Keeffe S, Midgley N. Developing Typologies in Qualitative Research: The Use of Ideal-type Analysis. Int J Qual Methods. 2022 Apr 1;21:16094069221100633.](https://www.zotero.org/google-docs/?Td9zY9)

[16.	Palinkas LA, Horwitz SM, Green CA, Wisdom JP, Duan N, Hoagwood K. Purposeful Sampling for Qualitative Data Collection and Analysis in Mixed Method Implementation Research. Adm Policy Ment Health Ment Health Serv Res. 2015 Sep 1;42(5):533–44.](https://www.zotero.org/google-docs/?Td9zY9)

[17.	United Nations Department of Economic and Social Affairs, Population Division. World Population Prospects 2022 - Documentation [Internet]. 2022 [cited 2024 Apr 15]. Available from: https://population.un.org/wpp/Download/Documentation/Documentation/](https://www.zotero.org/google-docs/?Td9zY9)

[18.	NTI, Johns Hopkins Bloomberg School of Public Health. Global Health Security Index: Building Collective Action and Accountability. 2019.](https://www.zotero.org/google-docs/?Td9zY9)

[19.	Sherratt K, Gruson H, Grah R, Johnson H, Niehus R, Prasse B, et al. Predictive performance of multi-model ensemble forecasts of COVID-19 across European nations. Wesolowski A, Ferguson NM, Shaman JL, Pei S, editors. eLife. 2023 Apr 21;12:e81916.](https://www.zotero.org/google-docs/?Td9zY9)

[20.	Ferguson N. UK COVID-19 Inquiry INQ000185337 - Witness Statement of Professor Neil Ferguson Module 1, dated 11/05/2023 [Internet]. 2023 Jul [cited 2024 May 21]. Available from: https://COVID19.public-inquiry.uk/documents/inq000185337-witness-statement-of-professor-neil-ferguson-dated-11-05-2023/](https://www.zotero.org/google-docs/?Td9zY9)

[21.	Jit M. How modelling can better support public health policy making: the Lancet Commission on Strengthening the Use of Epidemiological Modelling of Emerging and Pandemic Infectious Diseases. The Lancet [Internet]. 2023 Dec 20 [cited 2024 Feb 16];0(0). Available from: https://www.thelancet.com/journals/lancet/article/PIIS0140-6736(23)02758-7/fulltext](https://www.zotero.org/google-docs/?Td9zY9)

[22.	United Nations Department of Economic and Social Affairs, Population Division. World Population Prospects - Demographic Indicators [Internet]. 2022 [cited 2024 Apr 15]. Available from: https://population.un.org/wpp/Download/Standard/MostUsed/](https://www.zotero.org/google-docs/?Td9zY9)
