# Supplementary material for: How does policy modelling work in practice? A global analysis on the use of epidemiological modelling in health crises
Source: PLOS Glob Public Health. 2025 Jun 6;5(6):e0004675. doi: 10.1371/journal.pgph.0004675 (PMC12143527; doi:10.1371/journal.pgph.0004675)
Supplement: Attachment — Submitted filename: Response to reviewers.docx [file pgph.0004675.s005.docx]

Reviewer Comments and Revisions

“How does policy modelling work in practice? A global analysis on the use of epidemiological
modelling in health crises” by Hadley et al. (PGPH-D-25-00077)

We thank the reviewers and editor for their insightful comments that have helped strengthened our manuscript, particularly the reporting of qualitative methods. Please find our response to each reviewer/editor comment below.

| **Reviewer 1 comments** | **Action taken in revised manuscript** |
| --- | --- |
| Overall comments: *The manuscript addresses an important and timely topic concerning the role of epidemiological modelling in shaping public health decisions during crises. The study is well-structured, systematically conducted, and presents valuable insights from a diverse range of global experiences. The qualitative approach is appropriate given the study’s objectives, and the findings contribute significantly to ongoing discussions about best practices for integrating modelling into policymaking. However, there are areas requiring improvement in methodological clarity, analytical depth, and interpretation of findings.* | |
| Major comments:   1. *While the study employs a qualitative approach, the manuscript lacks sufficient justification for key methodological choices.* | Further details and justifications have been added to the manuscript Methods section and Supplementary Material to ensure clarity of approach and reproducibility. Please see individual actions taken in response to comments 2-13 below as well. Readers wishing to know more extensive information about the study are now also directed (in line 100-101 of the Revised Manuscript with Track Changes) to the lead researcher’s PhD thesis (forthcoming). |
| 2. *The authors mention stratified random sampling for the first country in each region, followed by purposive sampling. However, more details on how this selection was operationalised are needed.* | Text has been added to section 2.2 (‘Country and participant selection’) to fully explain the sampling procedures. |
| 3. *It is unclear whether participants had equal representation across the different countries or whether biases exist in the selection of decision-makers versus modellers.* | This data has been added to section 2.2 (lines 146-151). Specific countries are not named to maintain the anonymity of interviewees in countries with few modelling/policy actors. |
| 4. *The authors mention thematic and ideal-type analysis but do not provide sufficient detail on the coding framework or how themes were derived. The authors should provide more transparency on sampling and participant selection criteria and elaborate on the coding process and analytical approach.* | Two paragraphs have been added to section 2.4 (‘Data collection and analysis’) to describe in detail how the thematic and ideal-type analysis were carried out. Additional information has been added to the Supplementary Material S5 to further expand on the derivation of the typology. For sampling and participant selection criteria, text has been added to section 2.2 (‘Country and participant selection’). |
| 5. *The classification framework for modelling-policy systems is a strength, but its development lacks clear grounding in established typology methodologies.* | As noted above, the derivation in the Supplementary Material (now S5) has been rewritten for clarity, adding details on each step and its grounding in the established ideal-type analysis method of Stapley et al.. Further supporting references on typology literature are also supplied in S5 and in the main text (2.4). |
| 6. *The five identified categories appear descriptive rather than analytically derived. How were the key distinguishing factors determined? The authors should justify the classification framework with references to typology literature and provide explicit criteria used to assign countries to each category.* | See above. Determination of key features is now noted at the beginning of the Results, and described in detail in Supplementary Material S5. The explicit criteria used to assign countries to each category is described in Results section 3.1 and Supplementary Material S3, and its use in the subsequent typology has been highlighted in Results section 3.2 and Supplementary Material S5. |
| 7. *The authors make broad claims about the implications of their findings for global health crises. However, given the study's sample size (27 participants), caution is needed in extending conclusions beyond the selected countries. The exclusion of countries where modelling was not integrated into COVID-19 response introduces potential selection bias, as lessons could also be drawn from those settings. It will be prudent for the authors to acknowledge the limitations of sample size and selection bias and discuss the extent to which findings can be applied to other settings.* | Wording has been tweaked throughout to avoid overstating the implications of our findings, and more clarity has been given on the applicability of findings in the Discussion. The limitations of sample size and selection bias are acknowledged in lines 524-541 and also highlighted in lines 492-494 and 539-541. |
| Minor comments:   8. *The manuscript inconsistently uses terms like “modellers,” “scientific advisors,” and “decision-makers.” A clearer distinction of roles would improve comprehension. Also, the term “modelling-to-policy systems” is introduced but not consistently used throughout.* | Inconsistent uses of these terms have been removed to avoid confusion. Epidemiological modelling has also now been defined in lines 49-52, and more clarity is given on the different roles in lines 68-69 with signposting to the principal eligibility criteria in S1. Example job titles for the specific policy/ decision makers and science advisors included in our study are also provided in lines 151-154 and Table M2. |
| 9. *The manuscript presents policy uptake of modelling as primarily a structural issue but does not sufficiently explore political, economic, or cultural factors that may influence decision-making.* | This was outside the scope of our article and has been acknowledged as a limitation in lines 534-535. |
| 10. *The guidelines for future practice are useful but could be strengthened with more actionable steps.* | Text has been added to the Discussion to describe how the classification framework and guidelines for future practice could be implemented (lines 548-584). |
| 11. *The recommendation on interdisciplinarity acknowledges the need for collaboration with economics and behavioural sciences but does not suggest concrete strategies for achieving this.* | There was unfortunately limited data on this topic so concrete strategies could not be identified beyond providing simple case studies. This is acknowledged in Table R3. |
| 12. *Figure R1 (modelling infrastructure classification) is useful but would benefit from clearer labels distinguishing between different types of modelling structures.* | Clearer labels have been used and two minor phrases added to figure legend for clarity. |
| 13. *Table R1 (summary of country systems) is informative but dense. A more visual representation (e.g., colour coding or summary statistics) could improve readability.* | Excellent idea. Bold text and colour coding has been used on the key statistics in columns 2 and 6 to aid readability, and grey shading has been used on column 1 (country names). The names used in column 2 have also been streamlined with Figure R1 to aid comprehension. The authors also trialled adding in the illustrations used in Figure R1 to column 2 but this led to overcrowding. |

| **Reviewer 2 comments** | **Action taken in revised manuscript** |
| --- | --- |
| Overall comments:  *The manuscript titled "How does policy modelling work in practice? A global analysis on the use of epidemiological modelling in health crises" is original and first of its kind in the spectrum of epidemiological modelling analysis during global crises, with the COVID-19 pandemic being the case study.*  *The results were appropriate, and the discussion was also relevant.*  *This is a magnificent manuscript that could be accepted for publication if all the aforementioned concerns were addressed at the end of the day.* | |
| Major comments:   1. *Although the introduction was precise and concise, it did not fully address the modelling justification for future occurrences.* | Text and supporting references have been added to illustrate the justification of modelling in future disease control (lines 49-51 and 54-56). |
| 2. *There is a need for clarity of the peculiarities of each country in each global geographical region in terms of categories of Low-, middle- and high-income; multidisciplinary/multisectoral, hybrid versus streamlined discipline; and government involvement, Public-Private Partnership and private (institutional) approaches in the methods.* | The income status of each country, population size, and pre-pandemic preparedness ratings are highlighted in Table M1. Additional data on the peculiarities of each country such as level of multidisciplinary/ multi sectoral activity etc. was unfortunately not readily available. This has been acknowledged in the Methods section and readers are directed to a related study Dabak et al. (in preparation) that investigates public-private partnerships in more detail for some of the countries in our study. See text on lines 189-194. |
| 3. *More comparisons were required for aspects conducted during the non-crisis period in some parts of the geographical regions, like Europe and Asia.* | The focus of this study was crisis response, hence there is limited exploration of non-crisis periods. However we have added examples on non-crisis periods (post-COVID-19) in lines 557-570 and 573-575.  Note also that data analysis was deliberately not carried out by geographic region as vast differences were observed between countries in the same region - this is now illustrated in lines 480-494. |
| Minor comments:   4. Reviewer 2 also provided an attachment with minor suggestions to the manuscript grammar and punctuation. | These suggestions have been taken on board. Manuscript was written in the present tense and has now been changed to past tense. |

| **Editor’s comments** | **Action taken in revised manuscript** |
| --- | --- |
| *In addition to the reviewers suggestions, please address these points:*  1. *Please rework the first paragraph of the Introduction so it does not start with "This study examines the organisation of epidemiological modelling in Covid-19 response in 13 different*  *47 countries and jurisdictions." This sentence could be moved to the paragraph starting with "Our own study builds on this research gap..."* | Change has been made. |
| 2. *The section "Data collection takes the form of in-depth semi-structured interviews with national decision-makers, science advisors, and epidemiological modellers in 13 different countries. Interviewees span all six UN geographic regions, and capture the breadth of Covid-19 experience." belongs to the methods.* | These two sentences have been moved to the beginning of the Methods. |
| 3. *At the start of the Methods section, please add a "Study design" subheading and specify the design of the study. Did you use a qualitative descriptive design?* | This is a qualitative descriptive study. A ‘Study design’ section has been added (section 2.1). |
| 4. *Since this is a qualitative study, it would be worth reviewing the key points outlined in the Consolidated criteria for reporting qualitative research (COREQ) and make sure these are covered in the study.* [*https://www.equator-network.org/reporting-guidelines/coreq/*](https://www.equator-network.org/reporting-guidelines/coreq/) | Thank you for highlighting this criteria. We have added details throughout the Methods section to cover each point of the COREQ reporting criteria. |
| *Journal requirements:*  1. *Please include a complete copy of PLOS’ questionnaire on inclusivity in global research in your revised manuscript. Our policy for research in this area aims to improve transparency in the reporting of research performed outside of researchers’ own country or community. The policy applies to researchers who have travelled to a different country to conduct research, research with Indigenous populations or their lands, and research on cultural artefacts. The questionnaire can also be requested at the journal’s discretion for any other submissions, even if these conditions are not met. Please find more information on the policy and a link to download a blank copy of the questionnaire here:* [*https://journals.plos.org/globalpublichealth/s/best-practices-in-research-reporting*](https://journals.plos.org/globalpublichealth/s/best-practices-in-research-reporting)*. Please upload a completed version of your questionnaire as Supporting Information when you resubmit your manuscript.* | This form has been completed and has been uploaded as Supporting Information in our resubmission. |
| 2. *We note that your Data Availability Statement is currently as follows: “The available anonymised data has been presented in this manuscript and any associated manuscripts where possible.”*    *Please confirm at this time whether or not your submission contains all raw data required to replicate the results of your study. Authors must share the “minimal data set” for their submission. PLOS defines the minimal data set to consist of the data required to replicate all study findings reported in the article, as well as related metadata and methods (*[*https://journals.plos.org/plosone/s/data-availability#loc-minimal-data-set-definition*](https://journals.plos.org/plosone/s/data-availability#loc-minimal-data-set-definition)*).*    *For example, authors should submit the following data:*    *- The values behind the means, standard deviations and other measures reported;*  *- The values used to build graphs;*  *- The points extracted from images for analysis.*    *Authors do not need to submit their entire data set if only a portion of the data was used in the reported study.*    *If your submission does not contain these data, please either upload them as Supporting Information files or deposit them to a stable, public repository and provide us with the relevant URLs, DOIs, or accession numbers. For a list of recommended repositories, please see* [*https://journals.plos.org/plosone/s/recommended-repositories*](https://journals.plos.org/plosone/s/recommended-repositories)*.*    *If there are ethical or legal restrictions on sharing a de-identified data set, please explain them in detail (e.g., data contain potentially sensitive information, data are owned by a third-party organization, etc.) and who has imposed them (e.g., an ethics committee). Please also provide contact information for a data access committee, ethics committee, or other institutional body to which data requests may be sent. If data are owned by a third party, please indicate how others may request data access.* | Our Data Availability Statement is correct. Our submission does not contain all the raw data required to replicate the results, as unfortunately there is a very high identifiability and confidential sensitive information in both the transcripts and theme files. Sharing further raw data would compromise our ethical approval and participant consent was not sought for this. However, data requests may be sent to the [University of Cambridge Psychology Research Ethics Committee](https://www.bio.cam.ac.uk/psyres) (by emailing [SBSEthics@admin.cam.ac.uk](mailto:SBSEthics@admin.cam.ac.uk) and referencing application number PRE.2023.034), who can advise on this issue. |
